# The role of vitamin D in the severity and control of asthma in children and adolescents: A protocol for systematic review and meta-analysis

**DOI:** 10.1097/MD.0000000000031457

**Published:** 2022-12-16

**Authors:** Joelia Maria Costa Dias Ladeira, Olívia Zacas, Amanda Miranda Ferreira, Patrícia Chaib Gomes Stegun, Milena Baptistella Grotta, Adyleia Aparecida Dalbo Contrera Toro

**Affiliations:** a Department of Pediatric, State University of Campinas (UNICAMP), Campinas, São Paulo, Brazil.

**Keywords:** adolescent, asthma, children, vitamin D

## Abstract

Vitamin D plays an important role in the immune system and consequently in the inflammatory process of asthma. It acts directly on the regulation of helper T lymphocytes 1 (Th1) and helper T lymphocytes 2 (Th2) cells, and regulatory T lymphocytes. Evidence shows that vitamin D acts on dendritic cells, raising inflammatory mediators and increasing the imbalance between Th1, Th2, and helper T lymphocytes 17 (Th17). Evidence shows a strong association between vitamin D levels and asthma incidence, especially in patients with severe and uncontrolled asthma.

**Methods:**

A systematic and comprehensive search will be performed using four main databases, PubMed, EMBASE, Cochrane Library, and Web of Science. Articles will be searched from the earliest available time to august 2022. The studied population will be composed of children and or adolescents with asthma. From the data obtained, all articles found will be transferred to the Rayyan platform. Study selection will follow the Preferred Reporting Items for Systematic Reviews and Meta-Analysis Checklist (PRISMA P-2020). In addition, if sufficient data are available, a meta-analysis will be conducted. Two independent reviewers will conduct the studies selection, data extraction, and risk of bias assessment. The outcome measures will be to analyze the serum levels of vitamin D in patients with asthma and to relate this hormone to the control and severity of the disease and its anti- inflammatory effect.

**Results:**

A systematic review will provide better knowledge regarding vitamin D and its role in the severity and control of asthma.

**Conclusions:**

The findings of this study will be published in a peer-reviewed journal.

## 1. Introduction

Asthma is the most common chronic illness among children. Considered as a heterogeneous syndrome, it is characterized by chronic airway inflammation and a history of respiratory symptoms such as wheezing, shortness of breath, chest tightness, and cough that vary both over time and in intensity, along with variable expiratory airflow limitation.^[1]^ This heterogeneity is related to the phenotype of each patient, which is defined as the set of observable properties of the organism added to genetic and environmental conditions.^[1–3]^ It is associated with clinical and biological aspects, as well as molecular, cellular, and functional characteristics.

Vitamin D deficiency is also associated with asthma. The protective effects of vitamin D on asthma can be attributed to its immunomodulatory properties.^[4]^ Its relationship with asthma has been the focus of interest in recent years owing to its interference in fetal lung development, regulation of the balance of helper T lymphocytes 1 (Th1) and helper T lymphocytes 2 (Th2) cells, and maintenance of the number and functions of regulatory T lymphocytes (Treg T) cells.^[5]^ Evidence shows that vitamin D deficiency interferes with the maturation of dendritic cells, increases inflammatory mediators, and stimulating Th2 production. At normal levels, it can inhibit Th1 and Th2 responses and stimulate Treg T cells to secrete interleukin 10 (IL10) and transforming growth factor beta (TGF-β).^[6]^ It can also act on helper T lymphocytes 17 (Th17) lymphocytes by inhibiting the secretion of interleukin17, which has been associated with the presence of severe asthma.^[7]^

Genetic evidence links several genes connected to asthma that can be regulated by vitamin D and polymorphisms in its receptors, which may be related to an increased risk of attacks.^[8,9]^

Furthermore, vitamin D reduces hypertrophy in bronchial smooth muscle, hyperplasia of goblet cells, subepithelial deposition of collagen, and fibroblast activity, leading to a decrease in the asthma remodeling process.^[10]^

Several articles and evaluations in this field have shown that insufficient levels of vitamin D are associated with the exacerbation of asthma attacks in children and adolescents.

This systematic review will aim to identify, select, and appraise the vitamin D in the control and severity of asthma.

## 2. Methods

### 2.1. The registration

This systematic review protocol was registered with the International Prospective Register of Systematic Reviews (PROSPERO) in January, 2021 (registration number CRD42021221638). We will describe the changes in the full review, if necessary. This protocol report was prepared according to the guidelines described by Preferred Reporting Items for Systematic Review and Meta-Analysis Protocols (PRISMA-P).^[6]^ Furthermore, we elaborated the guiding question of this review, to ensure a systematic search of scientific literature using the population, intervention, comparison, outcomes, and study Design (PICOS).

### 2.2. Eligibility criteria

#### 2.2.1. Types of studies.

This review will include all types of primary study designs, including randomized control trials, prospective observational case-control studies, and cohort studies, published in English.

Table 1 shows the inclusion and exclusion criteria will be used in the study screening, first by title and abstract and then by full text. Articles will be included if they were primary studies. The study population must be composed of children and or adolescents with asthma. Studies should investigate the role of vitamin D in asthma control and severity (Table 1).

**Table 1:** Inclusion and exclusion criteria.

Include	Exclude
Types of studies: randomized trials, observational studies (including cohort and case-control studies) and also secondary studies	Types of studies: case report and letter to editor
Population: patients between 2 and 18 years old, of all ethnicities and genders	Population: adults, pregnant women, elderly, animals or infants with congenital heart defects
Language: articles published in English	Language: non-English articles
Outcome: higher levels of vitamin D correspond with better control and less severity of asthma	Outcome: recurrent wheezing by other causes

#### 2.2.2. Type of participants.

We will include children and adolescents aged 2 to 18 years diagnosed with asthma, irrespective of gender and ethnicity. All participants must be diagnosed with asthma using clearly defined or internationally recognized criteria.

#### 2.2.3. Type of interventions.

Studies to be examined will include any article on serum vitamin D levels in children and adolescents with asthma. Those articles report on vitamin D levels and asthma control or severity and inflammation will be considered.

#### 2.2.4. Type of outcome measures.

The primary outcome: The main criteria are:

1. Vitamin D level and asthma control;
2. Vitamin D level and asthma severity.

The secondary outcome:

1. Vitamin D and its anti-inflammatory effect through the regulation of Treg T cells.

### 2.3. Data sources and search strategy

We will search the following electronic bibliographic databases: PubMed, BVS-BIREME, EMBASE, EBSCOhost, Scopus, Web of Science, ProQuest, and the Cochrane Library and will use the following search strategy: (Child OR Adolescent) AND Asthma AND (“Vitamin D” OR Cholecalciferol) AND (“Inflammation Mediators” OR Lymphocytes OR Biomarkers OR Interleukins) (Fig. 2).

**Figure 1.**
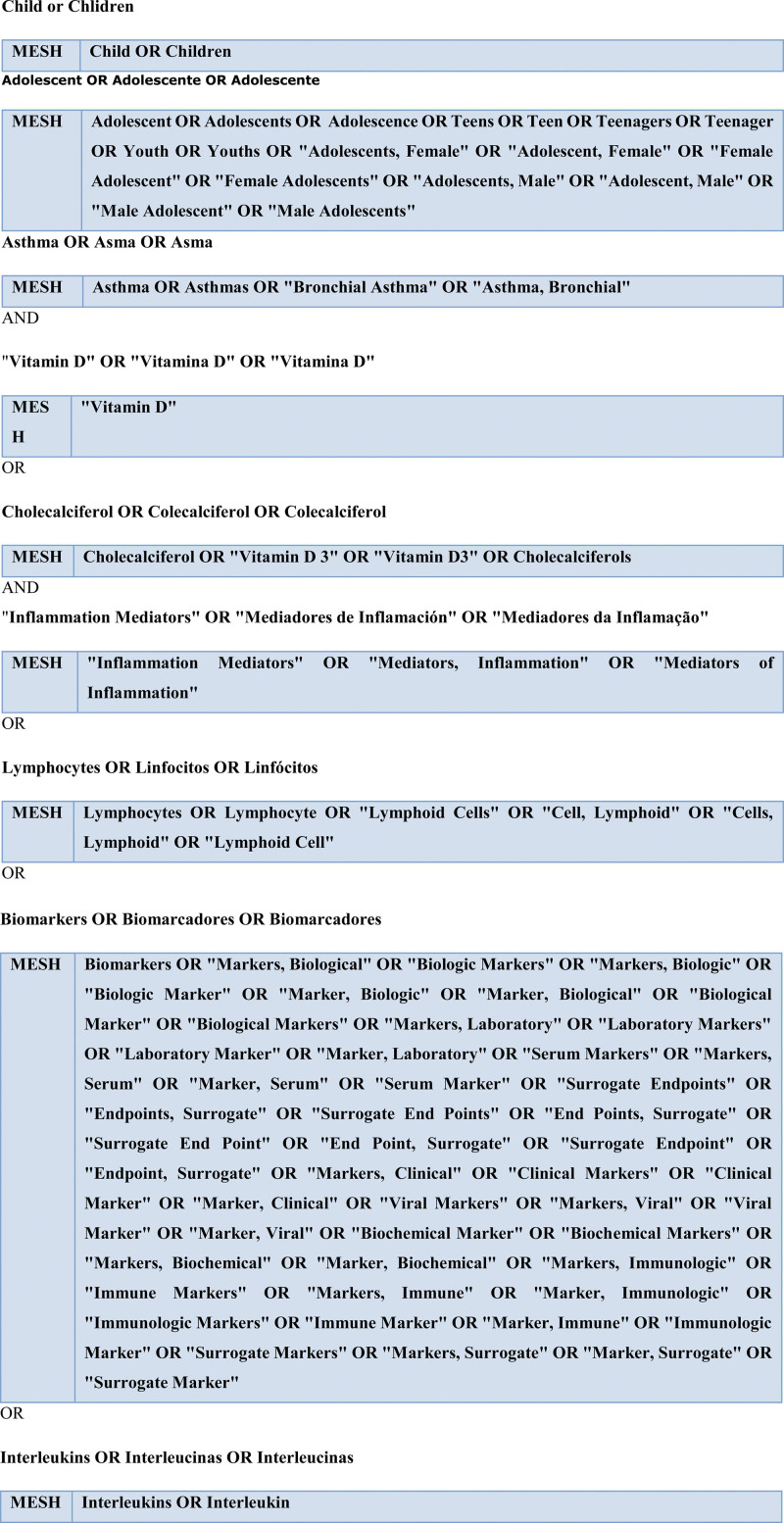
Preliminary search strategy.

**Figure 2.**
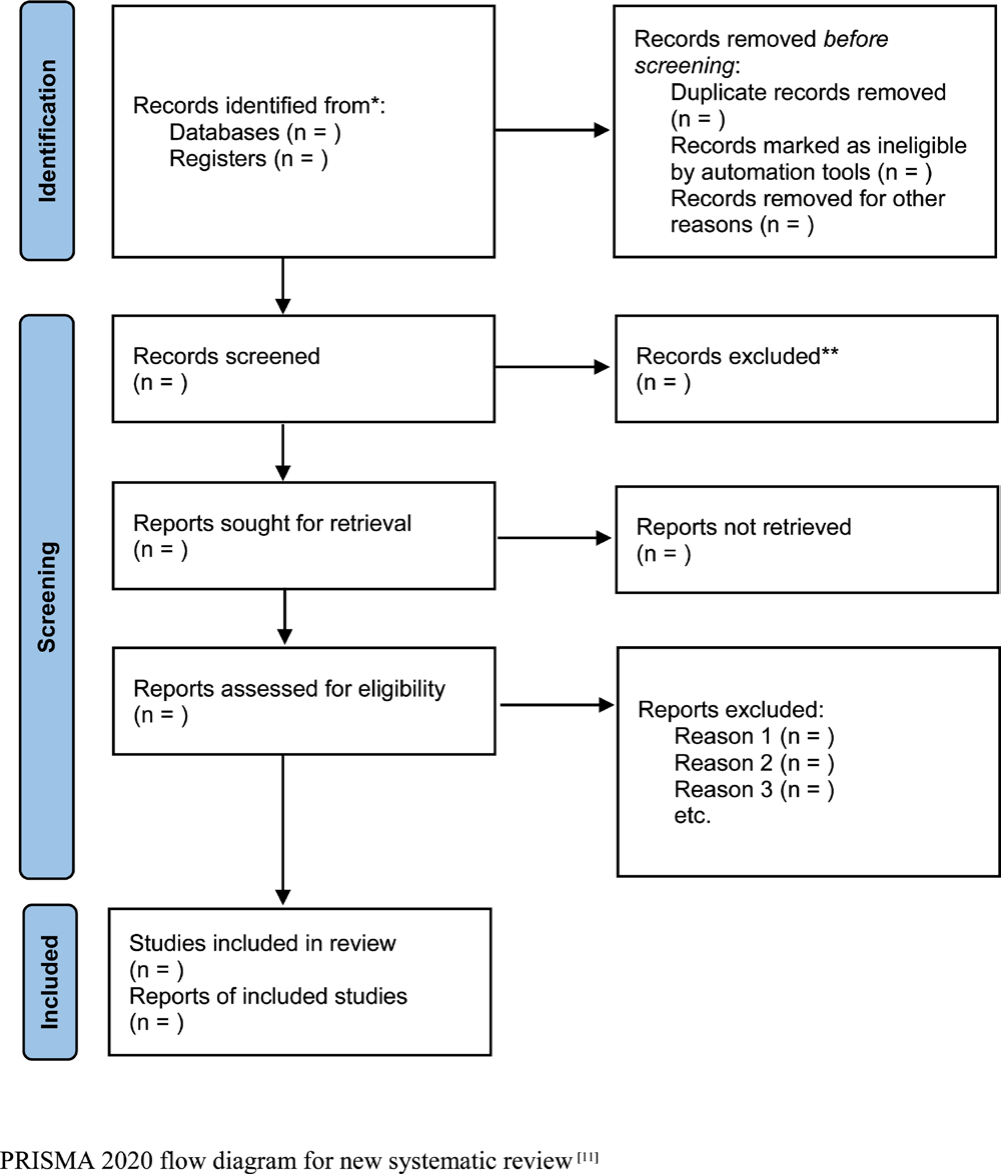
Information flow with different phases of a systematic review.

### 2.4. Data collection and analysis

#### 2.4.1. Study select and data selection.

The selected literature will be managed using Rayyan, and the screening will be a two-step process, first by title and abstract, and then by full text. A third investigator will be involved in cases of any disagreement or inconsistency that remains unresolved by consensus. Duplicate articles will be identified and excluded. To obtain qualified studies, we will then screen the full-text reports and decide whether they meet the inclusion criteria and then exclude studies with incomplete information. Details of the study selection procedure are presented in Figure 2.

#### 2.4.2. Data extraction and management.

Data from eligible articles will be independently extracted by two reviewers. If there are different opinions, we will discuss them. If any differences remain, a third reviewer will be consulted. The following data will be extracted: study characteristics, author names, year of publication, patient characteristics, data needed for quality assessment, and outcomes. If the data will be incomplete, it will be necessary to contact the original author. If data cannot be obtained the existing data should be transformed or the study should be excluded.

### 2.5. Assessment of risk of bias in included studies

The risk of bias for non-randomized studies will be assessed using the Newcastle–Ottawa scale (NOS), which assesses three parameters of study quality: selection, comparability, and exposure assessment. It assigns a maximum score of 4 for selection, 2 for comparability, and 3 for exposure, with a maximum total score of 9. Studies with a total NOS score of 5 or greater are considered to be of moderate to high quality, whereas those with an NOS score of less than 5 are considered low-quality studies. If we include randomized trial studies, we will assess their risks of bias with RoB 2.0 (a revised tool to assess the risks of bias in randomized trials). The quality of evidence for the clinical outcomes will be assessed according to the recommendations of the Grading of Recommendations Assessment, Development and Evaluation (GRADE) Working Group.

### 2.6. Data synthesis and statistical analysis

#### 2.6.1. Data synthesis.

RevMan 5.4 software will be used to conduct the meta-analysis. For dichotomous data, the vitamin D incidence will be reported as hazard ratios (RRs) with 95% confidence intervals (CIs). Statistical significance will be set at *P* < .05 for studies that present combinations of interventions and participants, a random effects model will be conducted.

#### 2.6.2. Assessment of heterogeneity.

Heterogeneity will be evaluated by the *I*² test. The value of *I*² ranges from 0% to 100% with 0% to 40% indicating no major heterogeneity, 40% to 60% indicating moderate heterogeneity, 60% to 90% indicating substantial heterogeneity, and >90% indicating considerable heterogeneity.

#### 2.6.3. Subgroup analysis.

Subgroup analysis proportion of asthmatic and non-asthmatic children with vitamin D deficiency, insufficiency, and sufficiency.

#### 2.6.4. Assessment of publication bias.

A funnel plot will be used to evaluate publication bias if more than 10 studies are included. RR from each study is plotted against their variance. Asymmetrical appearance of the plot indicates the presence of publication bias. Egger test will be used to test the asymmetry of the funnel plot.

#### 2.6.5. Sensitivity analysis.

Sensitivity analysis will be performed to assess whether the sample size and missing data affected the review results. If there are adequate studies (not less than three studies), we will perform a sensitivity analysis to verify the robustness of the conclusions and assess the impact of methodological quality.

#### 2.6.6. Confidence in cumulative evidence.

The quality of evidence will be assessed using the GRADE system. The evidence will be adjusted to 4 levels: high, moderate, low, or very low.

## 3. Discussion

Asthma is the most prevalent chronic respiratory disease in the world.^[11]^ If this review shows that vitamin D can help to control the disease and reduce the risk of severe asthma, it will support the hypothesis of a causal relationship between the level of vitamin D and the development of asthma. Furthermore, we hope that this study can support the premise about the biological mechanisms by which this might occur. This can have potentially enormous clinical implications, especially with the possibility to reduce asthma attacks, corticosteroid use, or/and hospitalization crises.^[11]^

Careful consideration of the research to date and future evidence-based recommendations will be needed to respond to patients’ concerns appropriately and precisely. Therefore, we planned this systematic review to summarize and assess the published evidence to date.^[12]^

One limitation of our study protocol is that many of the included studies may have poor methodological quality or may include insufficient explanation of their findings. Additionally, language bias may exist because only studies published in English will be considered due to language barriers.

## Acknowledgements

Mrs. Ana Paula Oliveira. Universidade Estadual de Campinas, Campinas SP, Brazil. José Dirceu Ribeiro, PhD. Department of Pediatric, State University of Campinas (UNICAMP), Campinas,São Paulo, Brazil.

## Author contributions

All authors contributed equally to this study. All authors approved the final version of the manuscript.

**Investigation:** Amanda Miranda Ferreira, Joelia Ladeira, Olívia Zacas, Patrícia Chaib Gomes Stegun.

**Methodology:** Amanda Miranda Ferreira, Joelia Ladeira, Olívia Zacas, Patrícia Chaib Gomes Stegun.

**Supervision:** Adyleia Aparecida Dalbo Contrera Toro, Milena Baptistella Grotta.

**Project administration:** Joelia Ladeira.

**Resources:** Olívia Zacas.

**Validation:** Joelia Ladeira, Milena Baptistella Grotta.

**Visualization:** Adyleia Aparecida Dalbo Contrera Toro, Amanda Miranda Ferreira, Joelia Ladeira, Olívia Zacas, Patrícia Chaib Gomes Stegun.

**Writing – original draft:** Amanda Miranda Ferreira, Joelia Ladeira, Olívia Zacas, Patrícia Chaib Gomes Stegun.

**Writing – review & editing:** Amanda Miranda Ferreira, Joelia Ladeira, Olívia Zacas, Patrícia Chaib Gomes Stegun.
